# Einfluss einer Mund-Nase-Maske auf die objektive körperliche Leistungsfähigkeit sowie das subjektive Belastungsempfinden bei gut-trainierten, gesunden Jungen

**DOI:** 10.1007/s10354-021-00851-9

**Published:** 2021-06-08

**Authors:** Benedikt Schulte-Körne, Wildor Hollmann, Argiris Vassiliadis, Hans-Georg Predel

**Affiliations:** 1 Institut für Kreislaufforschung und Sportmedizin, Deutsche Sporthochschule Köln, Am Sportpark Müngersdorf 6, 50933 Köln, Deutschland; 2 Olympiastützpunkt NRW/Rheinland, Guts-Muths-Weg 1, 50933 Köln, Deutschland

**Keywords:** Kinder, MNS, Körperliche Leistungsfähigkeit, RPE, Children, Surgical mask, Exercise performance, RPE

## Abstract

Mit dieser Studie wurden die Effekte chirurgischer MNS auf die körperliche Leistungsfähigkeit sowie das subjektive Belastungsempfinden bei trainierten elfjährigen Jungen untersucht.

Im aeroben und aerob-anaeroben Übergangsbereich fand sich keine Verminderung der objektiven Leistungsparameter. Allerdings zeigte sich auf der maximalen Belastungsstufe mit MNS eine signifikante Verminderung der Laufzeit sowie eine signifikante Erhöhung des subjektiven Belastungsempfindens bei ansonsten unveränderten Leistungsparametern. Zusammenfassend beeinflusst die Verwendung chirurgischer „Alltags“-MNS bei Kindern die sportlichen Aktivitäten nicht, solange die Belastungen primär im aeroben Intensitätsbereich durchgeführt werden.

## Abstract (en)

This study investigated the effects of surgical “everday” face masks on the physical performance and perceived exertion in trained eleven-year-old boys.

No decrease in objective performance parameters was found in the aerobic and aerobic-anaerobic transition zones. However, at the maximum performance level with surgical face mask, there was a significant reduction in running time as well as a significant increase in the subjective perception of exertion with otherwise unchanged performance parameters. In summary, the use of surgical face masks in trained children does not affect athletic activities as long as the loads are performed primarily in the aerobic intensity range.

## Hintergrund und Rationale

Im Zuge der aktuellen SARS-Covid-2-Pandemie haben sich handelsübliche dreilagige „chirurgische“ Mund-Nase-Schutzmasken (MNS) – neben Abstand und Hygieneregeln – als ein Instrument zur Bekämpfung der Verbreitung von SARS-CoV‑2 etabliert. Ihre konsequente Verwendung wird in vielen Berufs- und Alltagssituationen empfohlen bzw. verpflichtend vorgeschrieben [1]. Jedoch gibt es bzgl. der Verwendung von MNS im Rahmen sportlicher Aktivitäten, u. a. auch im Schulsport, bisher keine einheitlichen verbindlichen Empfehlungen [1]. Untersuchungen an Erwachsenen unter Verwendung von MNS zeigten keine signifikanten Effekte weder auf die körperliche Leistungsfähigkeit, Herzfrequenz (HF), Laktat, Sauerstoffsättigung (SpO_2_) noch auf das subjektive Belastungsempfinden (RPE) [2–7]. Jedoch zeigten sich negative Effekte auf die forcierte Vitalkapazität, den Peak Flow [2] und es wurde ein erhöhter Atemwegswiderstand beobachtet [7].

Bei Schulkindern hingegen finden sich bisher lediglich zwei Untersuchungen mit sog. „FFP2 (N95)“ -Masken, jedoch keine Studien zum Einfluss von „chirurgischen“ MNS [8]. Entsprechend liegen keine Daten zum Einfluss von MNS auf die körperliche/sportliche Leistungsfähigkeit sowie das RPE bei Schulkindern vor.

Ziel dieser Studie war es daher, den Einfluss von MNS auf die maximale körperliche Leistungsfähigkeit, RPE sowie HF, Plasma-Laktatkonzentration und SpO_2_ bei 11-jährigen männlichen Fußballspielern zu untersuchen.

Auf der Grundlage der bisher verfügbaren Studien bei Erwachsenen wurde die Hypothese postuliert, dass bei Schulkindern keine Beeinträchtigung der objektiven sportlichen Leistungsfähigkeit durch das Tragen einer MNS auftritt.

## Probanden und Methoden

### Probanden

11 männliche gesunde, trainierte Spieler der U12-Fußball-Mannschaft des 1. FC Köln (11,3 ± 0,3 Jahre, Größe: 149,8 ± 5,8 cm, Gewicht: 38,6 ± 4,4 kg) wurden in die Studie einbezogen. Der Trainingsumfang beträgt zusätzlich zum Schulsport vier wöchentliche Trainingseinheiten.

### Studiendesign

Prospektive, randomisierte, Cross-over-Studie.

### MNS (Mund-Nase-Schutzmaske)

Dreilagige MNS der Firma Medi-Inn (Frechen, Germany, zertifiziert: EN 14683:2019 (Typ II)).

### Untersuchungen

Für alle Probanden lag nach ausführlicher Information sowohl der Probanden als auch der Eltern eine schriftliche Einverständniserklärung der Eltern vor, weiterhin wurde die Studie von der lokalen Ethikkommission bewilligt. Jeder Proband absolvierte in randomisierter Reihenfolge auf dem Laufband (Firma H/P Cosmos sports & medical GmbH (München, Deutschland)) zwei Untersuchungen (einmal mit MNS, einmal ohne MNS) in einem für diese Altersgruppe etablierten stufenförmigen Belastungsschema (Startgeschwindigkeit 2,4 m/s, Stufendauer 3,0 min, Steigerung jeweils 0,4 m/s). Die beiden Belastungsuntersuchungen erfolgten in einem Abstand von exakt 7 Tagen, innerhalb derer die Probanden dem gewohnten Trainings- und Spielrhythmus nachgingen. Sämtliche Probanden wurden körperlich untersucht, die Körpertemperatur gemessen sowie der Peak Flow bestimmt. Alle Untersuchungen erfolgten unter Beachtung der aktuell gültigen Covid-19-Hygiene- und Infektionsschutzmaßnahmen.

Am Ende jeder Belastungsstufe wurden folgende Parameter ermittelt:

- Bestimmung der HF mittels Pulsgurt Polar T31 der Firma Polar Electro Europe AG (Steinhausen, Switzerland).
- RPE mittels standardisierter Borg-Skala (Gunnar Borg, 1982).
- Plasma-Laktatbestimmung mittels Abnahme von kapillärem Blut am Ohr (Auswertung: Biosen C‑Line Gerät, EKFdiagnostic GmbH, Barleben, Germany) und Bestimmung der Laktatschwellen (2 und 4 mmol/l) mit Hilfe des Computerprogrammes „winlactat“ der Firma Mesics GmbH (Münster, Germany).
- Messung der SpO_2_ mittels Finger-Pulsoxymeter PO-200 der Firma Pulox (Novidion GmbH, Köln, Germany).

### Statistische Analyse

Die angegebenen Ergebnisse wurden als Mittelwerte ± Standardabweichung dargestellt. Die weiteren statistischen Analysen (Shapiro-Wilk; Wilcoxon) wurden mit Hilfe von SPSS (IBM SPSS Statistics 27, Amonk, NY, USA) durchgeführt. Bei *p* < 0,05 wurde eine Signifikanz angenommen.

## Ergebnisse

**Figure 1:**
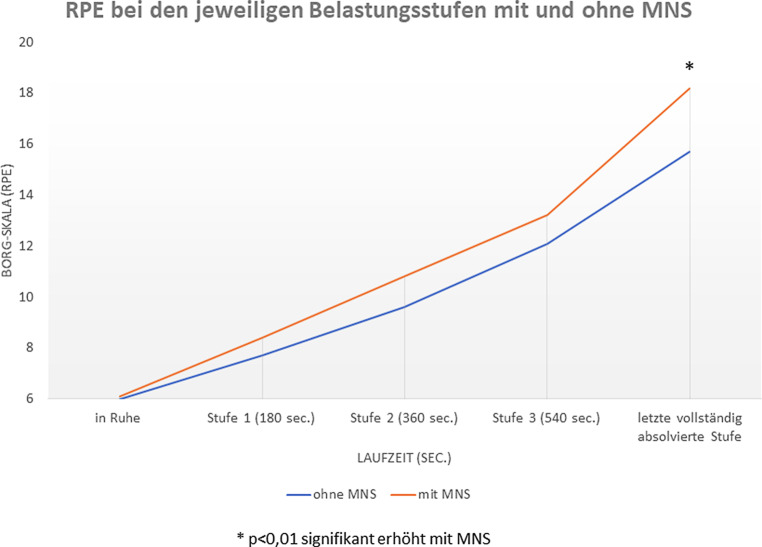

In beiden Laufbanduntersuchungen (mit und ohne MNS) fanden sich bzgl. der Laufgeschwindigkeit (km/h) sowohl im aeroben (2,0 mmol/l Laktat) als auch im aerob-anaeroben (4,0 mmol/l Laktat) Übergangsbereich keine signifikanten Unterschiede. Weiterhin zeigten sich auf allen Belastungsstufen für die Laktatkonzentration, die HF sowie die SpO_2_ mit und ohne MNS keine signifikanten Veränderungen (Tab. 1). Im Gegensatz dazu konnte während der stufenförmigen Belastungsuntersuchungen mit MNS eine signifikante Verminderung der Gesamtlaufzeit (808,6 ± 142,2 sec. vs. 914,6 ± 131,0 sec.; *p* = 0,001, siehe Tab. 1) beobachtet werden. Auf der maximalen Belastungsstufe konnte zudem eine signifikante Erhöhung des subjektiven Belastungsempfindens (RPE: 18,2 vs. 15,7 BORG-Skala; *p* = 0,007, siehe Abb. 1) mit MNS dokumentiert werden.

**Table 1:** 

	Ohne MNS (oMNS)	Mit MNS (mMNS)	oMNS vs. mMNS (*p*-Wert)
*Höchste vollständig absolvierte Belastungsstufe*			
Herzfrequenz (bpm)	189	191,4	0,36
Laktat (mmol/l)	4,45	4,15	0,49
SpO_2_ (%)	97,2	97,8	0,17
Borg-Skala	15,7	18,2	< 0,01*
*Laufgeschwindigkeit (km/h)*			
Bei 2,0 mmol/l Laktat	11,7	11,7	0,96
Bei 4,0 mmol/l Laktat	13,6	13,5	0,27
*Laufzeit (s)*			
Gesamtlaufzeit (s)	914,6	808,6	< 0,01*

*signifikant (*p* < 0,05)

## Diskussion und Schlussfolgerung

Angesichts der aktuellen Covid-19-Pandemie wird das Tragen von dreilagigen MNS (sog. „chirurgische Masken“) bei sportlichen Aktivitäten auch für Kinder, u. a. im Schulsport, diskutiert. Diese Diskussion wird bisher ohne das Vorliegen belastbarer wissenschaftlicher Untersuchungen zum Einfluss solcher MNS auf die körperliche/sportliche Leistungsfähigkeit von Kindern und Jugendlichen geführt. Mit dieser Studie wurden die Effekte dreilagiger MNS auf die körperliche Leistungsfähigkeit sowie das subjektive Belastungsempfinden im Rahmen einer stufenförmigen Laufbanduntersuchung bei elfjährigen gesunden Fußballern des 1. FC Köln evaluiert.

In dieser Untersuchung führte das Tragen einer MNS bei diesen gut-trainierten Jungen sowohl im aeroben (2 mmol/l Laktat) als auch im aerob-anaeroben (4 mmol/l Laktat) Übergangsbereich zu keiner Verminderung der objektiven Leistungsfähigkeit. Allerdings zeigte sich mit MNS im maximalen Belastungsbereich – entgegen unserer Ausgangshypothese – eine signifikante Verminderung der Gesamtlaufzeit und auf der maximalen Belastungsstufe eine signifikante Erhöhung des subjektiven Belastungsempfindens. Angesichts fehlender signifikanter Unterschiede auf dieser maximalen Belastungsstufe hinsichtlich Plasma-Laktatkonzentration, HF und SpO_2_ kann für die Laufzeitverminderung auf Basis der vorliegenden Daten keine eindeutige Ursache benannt werden. Auffallend ist jedoch, dass sich erst unter maximaler Belastung ein erhöhtes subjektives Belastungsempfinden der Probanden mit MNS manifestiert, welches am ehesten durch einen erhöhten Atemwegswiderstand durch die chirurgische MNS [2, 7] erklärt werden kann. Weiterhin kann auch ein verändertes Atemmuster, welches nicht Gegenstand dieser Untersuchung war, hierzu beigetragen haben [7, 9]. Auch ein „Nocebo-Effekt“ kann nicht ausgeschlossen werden.

Limitationen dieser Studie stellen das kleine Untersuchungskollektiv sowie das Fehlen weiblicher Probanden dar.

Zusammenfassend legen diese Beobachtungen nahe, dass die Verwendung einer „chirurgischen Alltags“-MNS für Kinder aus physiologischer Sicht auch bei sportlichen Aktivitäten grundsätzlich möglich ist, insbesondere solange die Belastungen primär im aeroben Intensitätsbereich durchgeführt werden. Allerdings sollte berücksichtigt werden, dass die maximale Leistungsfähigkeit bei Kindern durch die MNS negativ beeinflusst werden kann. Künftige, weiterführende Studien sollten größere Probandenkollektive und unterschiedliche kindliche Alterskohorten inklusive weiblicher Probanden einbeziehen.
